# Hand hygiene – social network analysis of peer-identified and management-selected change agents

**DOI:** 10.1186/s13756-019-0644-x

**Published:** 2019-11-28

**Authors:** Yew Fong Lee, Mary-Louise McLaws, Loke Meng Ong, Suraya Amir Husin, Hock Hin Chua, See Yin Wong, Didier Pittet, Walter Zingg

**Affiliations:** 1 Institute of Global Health, Faculty of Medicine, University of Geneva, Geneva, Switzerland; 2 Ministry of Health, Putrajaya, Malaysia; 3 School of Public Health and Community Medicine, UNSW Medicine, UNSW Sydney, Level 3 Samuels Building, Sydney, NSW 2052 Australia; 4 Clinical Research Centre & Department of Medicine, Hospital Pulau Pinang, Georgetown, Malaysia; 5 Sarawak General Hospital, Kuching, Sarawak Malaysia; 6 Infection Control Programme and WHO Collaborating Centre on Patient Safety, University of Geneva Hospitals and Faculty of Medicine, Geneva, Switzerland

**Keywords:** Hand hygiene, Alcohol-based handrub, Leadership, Social network analysis, Behavioural change, Multimodal strategy, Organizational culture

## Abstract

**Background:**

Hand hygiene compliance can be improved by strategies fostering collaborative efforts among healthcare workers (HCWs) through change agents. However, there is limited information about how change agents shape the social networks of work teams, and how this relates to organisational culture. The objectives of this study were to describe the influence of peer-identified change agents (PICAs) and management-selected change agents (MSCAs) on hand hygiene, perception of their leadership style by peers, and the role of the organisational culture in the process of hand hygiene promotion.

**Methods:**

This study, stratified in pre-, during, and post-intervention periods, was conducted between February 2017 and March 2018 in two wards at a tertiary care hospital in Malaysia. Hand hygiene promotion was facilitated either by PICAs (study arm 1) or MSCAs (study arm 2), and the two wards were randomly allocated to one of the two interventions. Outcomes were: 1) perceived leadership styles of PICAs and MSCAs by staff, vocalised during question and answer sessions; 2) the social network connectedness and communication patterns between HCWs and change agents by applying social network analysis; and 3) hand hygiene leadership attributes obtained from HCWs in the post-intervention period by questionnaires.

**Results:**

Hand hygiene compliance in study arm 1 and study arm 2 improved by from 48% (95% CI: 44–53%) to 66% (63–69%), and from 50% (44–55%) to 65% (60–69%), respectively. There was no significant difference between the two arms. Healthcare workers perceived that PICAs lead by example, while MSCAs applied an authoritarian top-down leadership style. The organisational culture of both wards was hierarchical, with little social interaction, but strong team cohesion. Position and networks of both PICAs and MSCAs were similar and generally weaker compared to the leaders who were nominated by HCWs in the post-intervention period. Healthcare workers on both wards perceived authoritative leadership to be the most desirable attribute for hand hygiene improvement.

**Conclusion:**

Despite experiencing successful hand hygiene improvement from PICAs, HCWs expressed a preference for the existing top-down leadership structure. This highlights the limits of applying leadership models that are not supported by the local organisational culture.

## Background

Globally, hundreds of millions of patients suffer from healthcare-associated infections every year with a higher burden in developing countries [1, 2]. Hand hygiene has been accepted as one of the most cost-effective measures in reducing both cross-transmission of microorganisms and healthcare-associated infections. Despite evidence of its effectiveness, compliance with hand hygiene is rather low [3]. This may be due to the nature of hand hygiene being an action, driven by behaviour [4], and taking part within the socio-economical and organisational context of healthcare facilities [5, 6]. Compliance is influenced by leadership engagement, peer pressure, and role modelling [5, 6]. Improvement strategies often fail because of insufficient implementation skills [7], which need to take into account behavioural aspects as part of the organisational culture [8, 9]. Strategies fostering collaborative efforts and the development of partnership among healthcare workers (HCWs) have been shown to be beneficial in both hand hygiene promotion and the prevention of healthcare-associated infections [5, 6, 10–13]. Such interventions aim at influencing social networks within work teams and across healthcare professions with the final goal to change the organisational culture of an institution [14]. There is limited information about the way change agents shape the social networks of work teams in healthcare, and how this relates to organisational culture.

The objectives of this study were to describe the influence of peer-identified change agents (PICAs) and management-selected change agents (MSCAs) on hand hygiene, the perception of their leadership style by peers, their ability to shape team dynamics, and the role of the organisational culture in this process. This report is part of a study examining the effect of change agents on hand hygiene behaviour in acute healthcare (Lee YF et al. Hand hygiene promotion delivered by change agents—Two attitudes, similar outcome, *forthcoming*).

## Methods

This study was conducted in collaboration with the Malaysian Ministry of Health, the World Health Organization (WHO) Collaborating Centre on Patient Safety at the University of Geneva Hospitals (HUG), Switzerland, and the University of New South Wales Sydney (UNSW Sydney), Australia. Malaysia is an upper-middle-income country in Southeast Asia [15]. Sarawak is the largest of the 13 states in Malaysia and is situated on Borneo Island [16]. The state has the most diverse population in Malaysia, with more than 40 ethnic groups, and the highest percentage of Christians [15, 16]. In recent years, the economy has shifted away from traditional mining, agriculture and forestry towards high-tech industries with renewable energy. Sarawak has 23 public hospitals and special medical institutions. The total number of public and private hospital beds per 1000 people was 1.5 in 2015 [17] compared with the average for Malaysia of 1.8 in 2012 [18]. The doctor-to-population ratio in Sarawak was 1:1184 [17] compared with the Malaysian average of 1:632 in 2017 [18].

### Setting

Between February 2017 and March 2018, two medical wards (study arm 1, study arm 2) were selected for an intervention on hand hygiene promotion at the Sarawak General Hospital, a university-affiliated, tertiary care hospital in Kuching, Malaysia. Kuching is the largest city and the economic centre in Sarawak with a total population of 325,132 [17]. The two wards were selected because of their interest, commitment and receptiveness towards improving hand hygiene behaviour (Lee YF et al. Hand hygiene promotion delivered by change agents—Two attitudes, similar outcome. *forthcoming*). Each ward had 42 official beds, with nurse-to-doctor ratios of 69:6 and 64:5, respectively. The nurse-to-patient ratio was 1: 2 in both wards. Both wards consisted of a mixed internal medicine adult patient population, often with chronic diseases and, in case of overflow in the intensive care unit, some patients underwent ventilation.

The study was stratified in pre-, during, and post-intervention periods of 48–56 days (Lee YF et al. Hand hygiene promotion delivered by change agents—Two attitudes, similar outcome. *forthcoming*).

### Intervention

Intervention, outcome measurement, and data entry are described in detail elsewhere (Lee YF et al. Hand hygiene promotion delivered by change agents—Two attitudes, similar outcome. *forthcoming*). In brief, before the pre-intervention period, HCWs in the two wards anonymously nominated and ranked 10 peers to become their change agents for hand hygiene promotion before the wards were randomly assigned to PICAs (study arm 1) or MSCAs (study arm 2). The nurse unit manager and the head of the medical department selected change agents for study arm 2 during the pre-intervention period. In total, six change agents, five nurses and 1 doctor, were selected for each study arm to assure that at least one change agent would be present on any study day. To reduce bias, no information on randomisation was revealed, and both PICAs and MSCAs were told that they were selected by senior management. Senior management refers to members of the medical and nursing boards of the department. The 6 PICAs in study arm 1 were HCWs with the highest numbers of nominations by their peers. Both PICAs and MSCAs were given the task to promote hand hygiene in their work teams during the intervention period by encouraging peers to perform hand hygiene, giving feedback, and offering correction or congratulation on missed or observed hand hygiene opportunities.

### Outcome measurement

Trained and validated auditors measured hand hygiene compliance by direct hand hygiene observation using the WHO methodology [19], and has been reported separately (Lee YF et al. Hand hygiene promotion delivered by change agents—Two attitudes, similar outcome. *forthcoming*). During the intervention period, the principal investigator conducted 8 walk-arounds in each study arm with the aim of observing interactions between change agents and staff and initiatives taken by the change agents to promote hand hygiene. In the post-intervention period, six question and answer (Q&A) sessions in both English and Malaysian were organized to explore the opinion of nurses towards PICAs and MSCAs. The sessions were conducted over 6 days, with a maximum of 10 randomly allocated participants per session, in total 57 and 55 nurses in study arm 1 and study arm 2, respectively. Written informed consent was obtained from all participants. A local infection prevention and control expert facilitated the Q&A sessions using a semi-structured interview guide that addressed five areas of leadership attributes: attitude, self confidence, approachability, team role, and decision-making capacities [5, 20]. The sessions were audio-recorded, transcribed verbatim and translated into English. In addition, the participating nurses re-nominated five peers (nurses only) they preferred as leaders for hand hygiene promotion. Doctors did not participate in this exercise. They also listed and ranked (as free text) three main leadership qualities they considered important for hand hygiene promotion.

### Social network analysis

Data from the re-nomination lists were used to perform social network analysis with the NodeXLPro software (Social Media Foundation, Belmont, CA, USA) [21]. Three dimensions were distinguished: 1) visual sociograms of the entire network; 2) calculations of geodesic distance, density, and reciprocity of the network as a whole; 3) calculation of centrality of the five most nominated individuals. Sociograms visualize the position of staff within the post-intervention re-nomination network. Each HCW on the ward represents a ‘node’ and the lines between the nodes describe the personal network of the HCWs and the relational distance within this network. Geodesic distance measures the shortest route or pathway between two individuals and the range of their connections within the network. The distance is reported as an average and maximum distance between individuals [22, 23]. Density measures the total interactions (or relationships) between individuals divided by the total possible interactions (relationships) between individuals within the network. Reciprocity measures the degree of reciprocal nominations between individuals (individuals nominating each other). Vertical hierarchies have low reciprocity (little nominations between individuals), while horizontal hierarchies have high reciprocity (many nominations between individuals). Centrality measures individuals who are most connected and as such will hold influential positions in the network [24]. The most common measures are degree centrality, closeness centrality, and betweenness centrality [25]. In the current study, degree centrality is the measurement of nominations directed towards an individual within the network [24, 25]. Closeness centrality is the average length of the shortest path between individuals. Closeness centrality captures how close one individual is to other individuals within the network, based on how quickly or easily each individual can interact with other individuals. Smaller numbers indicate shorter distance (easier interaction). Betweenness centrality measures the number of times an individual is on the shortest path between two other individuals. A high betweenness centrality indicates that an individual is an influential gatekeeper and is connected to the otherwise disconnected individuals in the network.

## Results

### Hand hygiene compliance

Compared to the pre-intervention period, hand hygiene compliance improved from 48% (95% CI: 44–53%) to 66% (63–69%), and from 50% (44–55%) to 65% (60–69%) during the intervention period in study arm 1 and study arm 2, respectively. There was no significant difference of hand hygiene improvement between the two study arms (Lee YF et al. Hand hygiene promotion delivered by change agents—Two attitudes, similar outcome. *forthcoming*).

### Question and answer sessions

Perceived leadership styles of PICAs and MSCAs, expressed by HCWs during the Q&A sessions in the post-intervention period, differed substantially between the two study arms and in all five areas of leadership attributes (Table 1). In accordance with observations during the ward walk-arounds, MSCAs were reported to apply an authoritative leadership style while PICAs acted more by being a role model.

**Table 1:** Perceived leadership styles of peer-identified change agents and management-selected change agents

Leadership attributes	Study arm 1 Peer-identified change agents	Study arm 2 Management-selected change agents
Attitude	Not pushy, mostly easy-going; positive attitude; motivated to improve hand hygiene practices in the ward	Very determined to improve hand hygiene compliance; strong-minded; goal orientated
Self-confidence	Somewhat lacking self-confidence; concerns about their leadership capacities	Very assertive
Approachability	Visible and always receptive; very friendly; not strict; empathetic	Approachable attitude, but sometimes unavailable due to other activities; were strict; almost dictatorial
Team role	Strong team players; followed-through tasks with other team members; always performed good hand hygiene practices (good role models)	Acted as team managers; kept their distance; always performed good hand hygiene practices (good role models)
Decision-making capacities	Somewhat lacking	Prominent

A total of 69 and 68 leadership attributes for hand hygiene promotion were received from Q&A participants of study arm 1 and study arm 2, with 201 and 172 citations, respectively. The most commonly quoted leadership quality for hand hygiene promotion was a “strict attitude”, cited by 40 HCWs (40/56; 70%) in study arm 1 and 27 times (27/55; 49%) in study arm 2 (Table 2).

**Table 2:** Preferred leadership qualities for hand hygiene promotion cited by healthcare workers

Study arm 1 (PICAs)				Study arm 2 (MSCAs)			
Rank	Preferred leadership qualities	Frequency (*n* = 56 HCWs)		Rank	Preferred leadership qualities	Frequency (*n* = 55 HCWs)	
		*N*	%			*N*	%
1	Strict	40	71.4	1	Strict	27	49.1
2	Responsible	29	51.8	2	Good role model	25	45.5
3	Hardworking	14	25.0	3	Always reminds	15	27.3
4	Knowledgeable	9	16.1	4	Responsible	14	25.5
5	Speaks up	9	16.1	5	Committed / dedicated	12	21.8
6	Disciplined	6	10.7	6	Friendly	9	16.4
7	Senior	6	10.7	7	Disciplined	8	14.5
8	Soft spoken	5	8.9	8	Hardworking	5	9.1
9	Honest	5	8.9	9	Good attitude	5	9.1
10	Good teacher	4	7.1	10	Good teacher	4	7.3

*HCWs* Healthcare workers, *PICAs* Peer-identified change agents, *MSCAs* Management-selected change agents

### Social network analysis

**Fig. 1:**
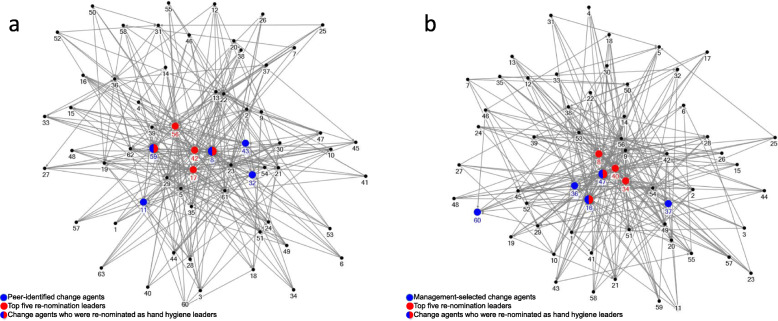
Sociograms visualizing the positions of peer-identified and management-selected change agents (5 nurses each) within the re-nominated leaders. **a** Study arm 1 – Peer-identified change agents. **b** Study arm 2 – Management-selected change agents

Social network analysis was performed on 56 and 55 HCWs in study arm1 and study arm 2, respectively. One HCW from study arm 1 did not participate in the social network questionnaire. Ward HCWs included senior, junior and auxiliary nurses with an average work experience of 7 years in study arm 1 and 6 years in study arm 2, respectively. The wards were similar for the number of nominated relationships with 315 and 295 in study arm 1 and study arm 2, respectively. The sociograms illustrate similar social networks in both study arms (Fig. 1). Peer-identified change agents and MSCAs who were re-nominated in the post-intervention period had strong networks. One PICA (#11) and one MSCA (#60) who were nominated in the pre-intervention period but not re-nominated in the post-intervention period were less connected than other initially nominated HCWs. Figure 1 shows the visual sociograms of the entire network.

The average and maximum geodesic distances between individuals in both study arms were 1.9 and 3.0, respectively. On average, each HCW in both study arms was only two steps away from a re-nominated leader, indicating that both networks were cohesive. The density of both networks was 0.08 for each study arm, indicating that there was little interaction between HCWs in both networks. Reciprocities of study arm 1 and study arm 2 were low, 0.08 and 0.10, respectively. This indicated a hierarchical network with only 8 and 10% of the pairs being nominated by each other.

The medians for the degree centrality, closeness centrality, and betweenness centrality of PICAs and MSCAs indicate that the leadership styles of both PICAs and MSCAs would equally influence hand hygiene (Table 3). Ranges of centrality were similar in both study arms.

**Table 3:** Centrality of the change agents nominated before the pre-intervention period

	ID	Change Agent	Centrality		
			Degree	Closeness	Betweenness
Study arm 1 PICAs	8	Yes	25	0.010	387.8
	59	Yes	18	0.010	234.3
	43	Yes	7	0.008	70.6
	11	Yes	7	0.008	46.7
	22	Yes	5	0.008	40.8
	Median		7	0.008	70.6
Study arm 2 MSCAs	47	Yes	25	0.011	327.4
	16	Yes	21	0.011	211.0
	36	Yes	12	0.010	86.5
	37	Yes	4	0.009	25.7
	60	Yes	3	0.009	18.8
	Median		12	0.010	86.5

*PICAs* Peer-identified change agents, *MSCAs* Management- selected change agents

The median centralities for the top five re-nominated leaders in the post-intervention period were similar in both study arms (Table 4). However, the range for the betweenness centrality in study arm 2 was wide (232.7–687.0), while this range in study arm 1 was narrow (234.3–387.8).

**Table 4:** Centrality of the top five re-nominated leaders in the post-intervention period

	ID	Change Agent	Centrality		
			Degree	Closeness	Betweenness
Study arm 1 PICAs	8	Yes	25	0.010	387.8
	17	No	23	0.010	371.7
	42	No	23	0.010	359.8
	56	No	24	0.010	315.0
	59	Yes	18	0.010	234.3
	Median		23	0.010	359.8
Study arm 2 MSCAs	40	No	36	0.013	687.0
	34	No	28	0.011	438.1
	47	Yes	25	0.011	327.4
	8	No	23	0.011	295.9
	16	Yes	21	0.011	232.7
	Median		25	0.011	327.4

*PICAs* Peer-identified change agents, *MSCAs* Management- selected change agents

Compared to PICAs, MSCAs had more work experience both overall and in the ward (Table 5). Both PICAs and MSCAs had low betweenness centralities compared to the top-five re-nominated leaders (Table 5).

**Table 5:** Work experience and centrality of change agents and the top-five re-nominated leaders

		Working years in healthcare Median (range)	Working years in the ward Median (range)	Centrality Median (range)		
				Degree	Closeness	Betweenness
Study arm 1 PICAs	Peer-identified change agents	5 (3–8)	5 (3–5)	7 (5–25)	0.008 (0.008–0.010)	70.6 (40.8–387.8)
	Top five re-nominated leaders	15 (3–17)	5 (2–15)	23 (18–25)	0.010 (0.010–0.010)	359.8 (234.3–387.8)
Study arm 2 MSCAs	Management selected change agents	19 (10–24)	8 (2–10)	12 (3–25)	0.010 (0.009–0.011)	86.5 (18.8–327.4)
	Top five re-nominated leaders	14 (6–24)	3 (2–9)	25 (21–36)	0.011 (0.011–0.013)	327.4 (232.7–687.0)

*PICAs* Peer-identified change agents, *MSCAs* Management- selected change agents

## Discussion

The fact that there was no difference in hand hygiene compliance between the two study arms was a major finding of the intervention strategy of the overall study (Lee YF et al. Hand hygiene promotion delivered by change agents—Two attitudes, similar outcome. *forthcoming*). Qualitative Q&A sessions provided in-depth insight into perceptions of leadership styles by HCWs, while quantitative social network analysis explained relationships of complex interactions in work teams. The methods complemented each other and provided a picture of socially cohesive work teams with staff nominating socially well-connected peers, while preferring the status-quo of a strict, even authoritarian, leadership style for hand hygiene promotion.

The little interaction between HCWs, the low number of re-nominated leaders (2/5), and the low nominator-nominee ties suggest that the networks in both study arms were highly hierarchical. The average short distance between the HCWs suggested that both work teams were cohesive. However, poor reciprocity and low ties (interactions) suggested that the HCWs did not rely on each other for assistance, at least not for hand hygiene.

Two of the five re-nominated leaders in both study arms had been identified as change agents. Their re-nomination supports the diffusion of innovation theory [26] hypothesis that change agents are early adopters who make change acceptable for their peers. In the context of facilitating the adoption of good hand hygiene practice behaviour, these change agents were important for linking disconnected HCWs towards hand hygiene practice.

The PICAs in study arm 1 promoted hand hygiene improvement through leading by example. However, HCWs in this study arm indicated that, when given a choice, they preferred authoritarian leadership, even though they also expressed unease with this leadership style, and they re-nominated rather socially skilled peers in the post-intervention period. These findings exemplify the predominance of the existing organisational culture on behaviour, even if it causes cognitive dissonance [9]. This may have been shaped by the local culture of native tribes in Sarawak always to appoint elderly seniors to be leaders [27]. However, preference for hierarchical organization structures by HCWs has been reported also in other countries and in different healthcare settings [28–30].

Our study has limitations. First, concerns about confidentiality in the Q&A sessions may have motivated HCWs to re-nominate leaders for hand hygiene promotion based on social expectations rather than their personal preference. Second, the study duration was relatively short to observe the full effect of a behavioural change intervention on organisational culture. A prolonged post-intervention period may have influenced both hand hygiene compliance and the perception of leadership style by staff. We hypothesise that in the long-term, the effect of PICAs on hand hygiene compliance may be superior compared to MSCAs, because HCWs feel more at ease working with them. Third, the limited, although significant, improvement of hand hygiene compliance exemplifies the reality given the short study periods and mandating internal change agents.

## Conclusion

Despite experiencing successful hand hygiene improvement from PICAs who led by example, HCWs expressed a preference for the existing authoritarian leadership structure. This highlights the limits of applying leadership models that are not supported by the local organisational culture, and urges the need to repeat such study in other cultural settings. There was no difference in hand hygiene improvement between both study arms.

## Data Availability

The datasets used and/or analysed during the current study are available from the corresponding author on reasonable request.

## Abbreviations

HCWs: Healthcare workers
MSCAs: Management-selected change agents
PICAs: Peer-identified change agents
Q&A: Question and Answer
WHO: World Health Organization
